# Retroperitoneal neuroglial heterotopia: a case report and literature review

**DOI:** 10.3389/fped.2024.1369787

**Published:** 2024-04-08

**Authors:** Jianhua Zhong, Lijun Yang, Jinhui Lin, Ruifa Wu, Wenguang Liu, Qinfang Xu, Da Ma, Zhibo Qu

**Affiliations:** ^1^ Department of Pediatrics, Dongguan Children’s Hospital, Dongguan, Guangdong, China; ^2^ Department of Pathology, Dongguan Children’s Hospital, Dongguan, Guangdong, China; ^3^ Department of Anesthesiology, The Fourth Affiliated Hospital of Guangzhou Medical University, Guangzhou, Guangdong, China

**Keywords:** neuroglial heterotopia, cystic mass, case report, laparoscopy, retroperitoneum

## Abstract

**Background:**

Neuroglial heterotopia is a rare lesion composed of differentiated neuroectodermal cells that manifest in extracranial locations, with the majority of cases predominantly occurring in the head and neck region. Retroperitoneal neuroglial heterotopia is exceptionally rare, with isolated cases published in the scientific literature.

**Case report:**

Here, we present the case of a 3-year-old girl who was admitted without clinical signs but presented with a palpable abdominal mass. Ultrasonography and computed tomography scans revealed a sizable cystic lesion within the retroperitoneal space. Subsequently, laparoscopic resection was performed. Histological examination unveiled neuroglial cell-lined cysts encompassing fibrous connective tissue, ganglia, glial tissue, and nerve bundles. Notably, distinct areas and cell types exhibited expression of S100, glial fibrillary acidic protein, and neuron-specific enolase. Follow-up assessments revealed no relapses or late complications.

**Conclusion:**

In cases of retroperitoneal neuroglial heterotopia, most children may remain asymptomatic without any congenital anomalies. Despite their detectability through imaging, accurate preoperative diagnosis is seldom achieved. Generally, a favorable prognosis follows complete surgical resection, although further cases are required to confirm its long-term efficacy, necessitating extended follow-up for verification.

## 1 Introduction

Neuroglial heterotopia (NGH) is a rare congenital anomaly characterized by the presence of cerebral tissue in extracranial locations (1). As non-neoplastic lesions, these are primarily located in the head and neck region, particularly the nose and nasopharynx (2). Other reported sites of occurrence include the oropharynx, middle ear, eyes, orbit, skull, tongue, lips, scalp, lung, skin, extremities, peritoneum, and ovaries (3–7). Since its initial description by Wan et al. (8), only five cases in the retroperitoneum have been documented (8–12). In this report, we present a further case involving a huge retroperitoneal neuroglial heterotopia that was successfully resected laparoscopically. The pathogenesis and clinical characteristics of previously published cases are summarized and discussed herein.

## 2 Case report

**Figure 1:**
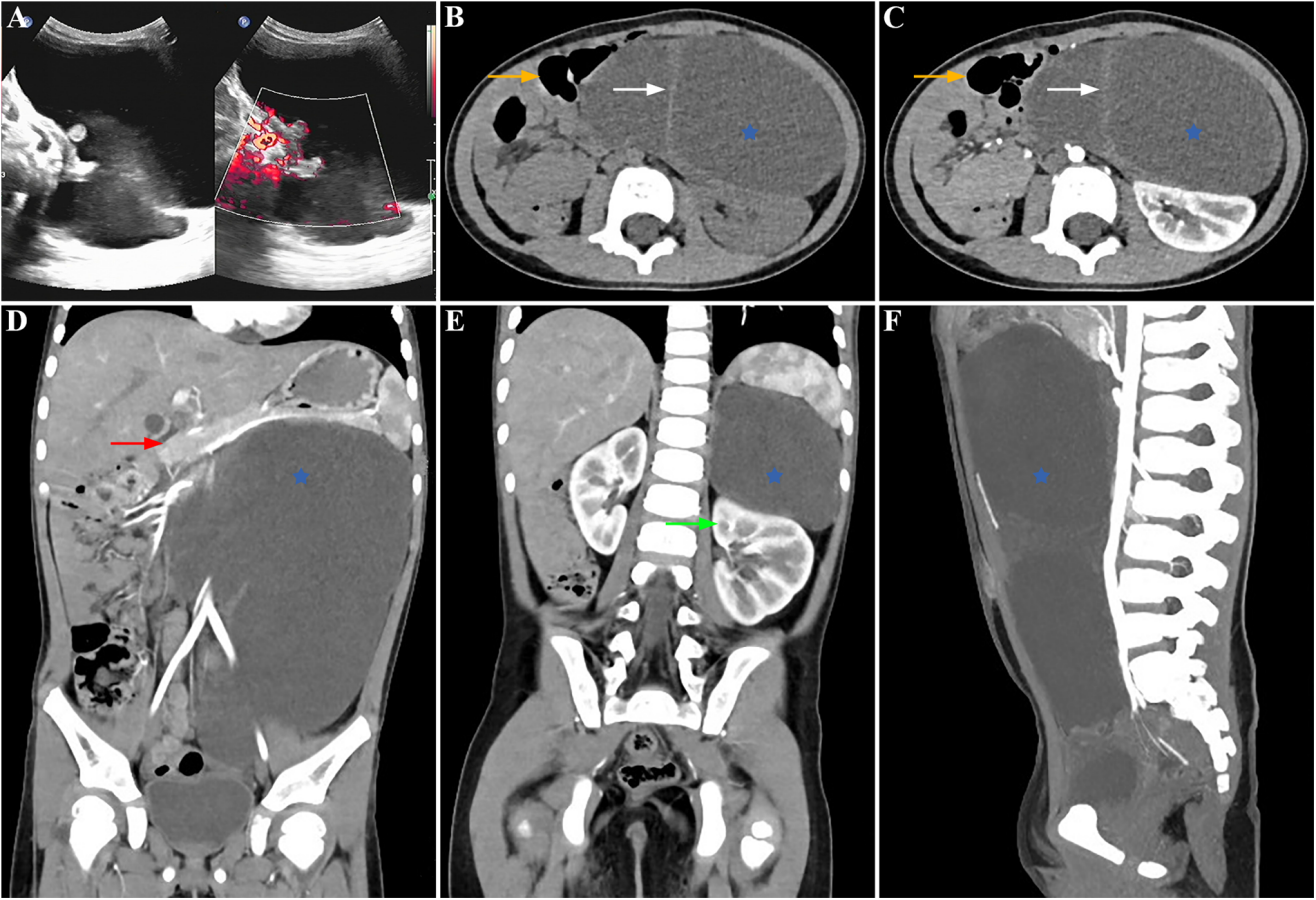
Preoperative findings: the lesion showed anechoic cysts with no obvious blood flow signal in the cyst wall in the left abdomen on an ultrasonogram (**A**). Contrast-enhanced abdominal-pelvic CT scanning showed a huge, slightly low-density lesion (blue star) with some septations (white arrow) in the retroperitoneum, which compressed the neighboring organs, pushing the left kidney downward (green arrow), pancreas upward (red arrow), and intestine rightward (orange arrow) (**B,C**: axial view, **D,E**: coronal view, **F**: sagittal view).

A palpable abdominal mass was identified in a 3-year-old asymptomatic girl (weight 16.8 kg, height 104.5 cm, BMI 15.4 kg/m^2^) during a pre-kindergarten admission physical examination and confirmed via ultrasound in the outpatient department. The patient had normal bowel movements and no history of urinary tract infections, unexplained fevers, hematuria, dysuria, weight loss, recent abdominal trauma, or surgery. There was no significant family history of cancer or neurological deficits. Clinical evaluation revealed a painless cystic mass measuring approximately 12 cm in diameter on the left flank. Rectal palpation did not reveal any abnormalities. While neuron-specific enolase (NSE) levels were slightly elevated (20.76 ng/ml), other tumor biomarkers (carbohydrate antigen 125, carbohydrate antigen 199, carcinoembryonic antigen, alpha-fetoprotein, human chorionic gonadotropin) were within normal ranges. Routine blood tests, blood biochemistry, and urinalysis showed no abnormal findings. The abdominal ultrasound indicated a large hypoechoic mass characterized by a thick wall, septations dividing cystic spaces, absence of mural nodules, and no blood flow signals on color Doppler flow imaging (Figure 1A). Intravenous contrast-enhanced abdominal-pelvic computed tomography (CT) imaging revealed a retroperitoneal cystic lesion measuring 135 mm × 117 mm × 186 mm (Figures 1C,D,F) without invading adjacent structures but displacing the left kidney downward (Figure 1E), pancreas upward (Figure 1D), and intestine rightward (Figures 1B,C). The capsule and septa showed slight enhancement, while the cystic components lacked enhancement (Figures 1B,C). Due to insufficient financial capabilities, magnetic resonance imaging (MRI) was not performed. The differential diagnosis included a cystic teratoma, bronchogenic cyst, or cystic lymphangioma, prompting further investigation through laparoscopy for definitive diagnosis and treatment.

**Figure 2:**
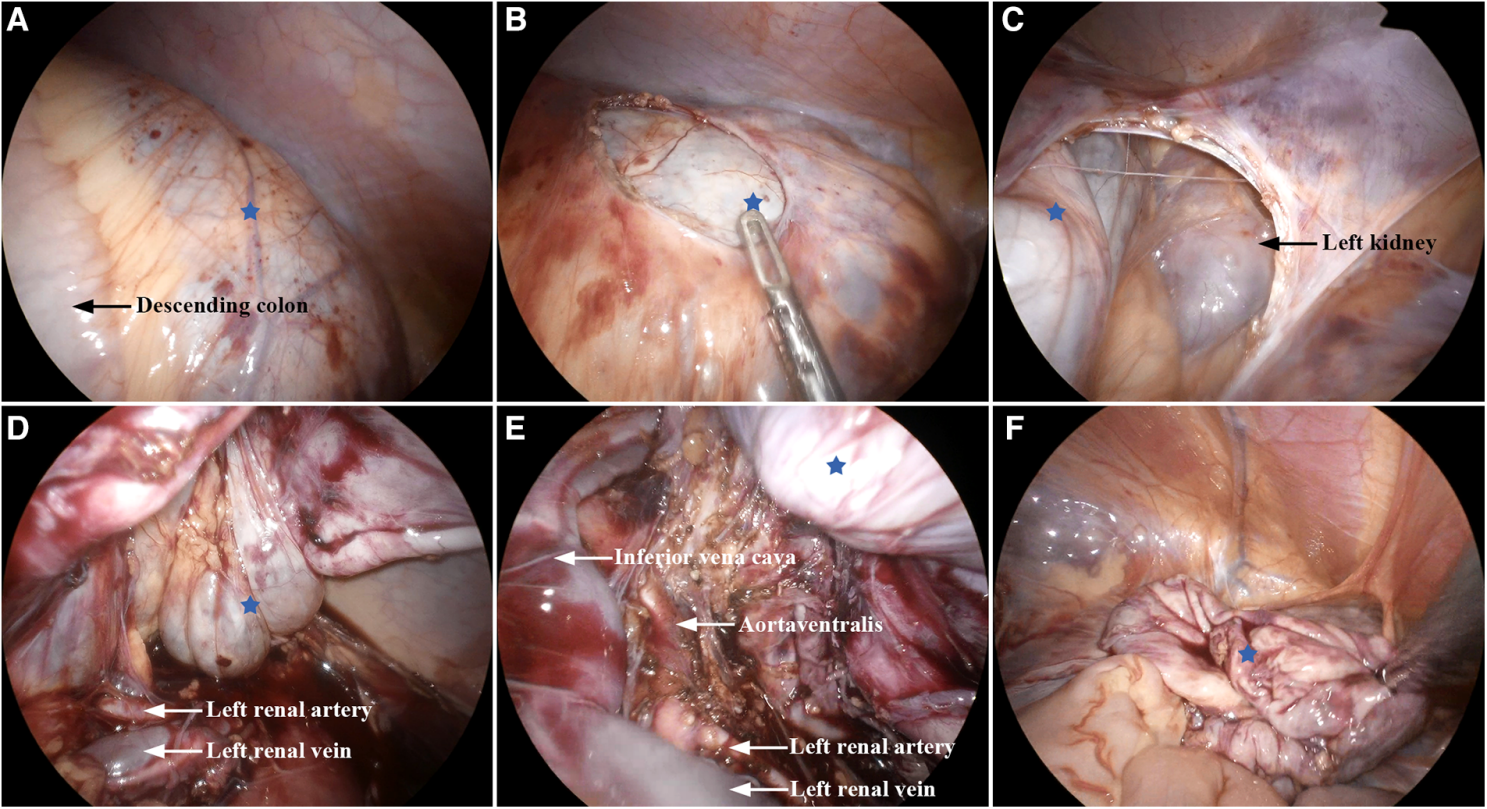
Intraoperative findings: (**A**) huge cystic lesion (blue star). (**B,C**) Retroperitoneal lesion located in the left anterior pararenal space. (**D,E**) Lesion adhered to the left renal vein, left renal artery, inferior vena cava, and aorta ventralis. (**F**) Lesion was removed completely.

Laparoscopic exploration was commenced using three 5-mm ports in the supine position. During laparoscopic observation, a large thick-walled retroperitoneal cyst was identified (Figure 2A), extending toward the spleen, adrenal gland, cauda pancreatis, and down to the left ovarian appendage. The cyst exhibited a complete capsule, regular morphology, and no signs of hemorrhage. An intraoperative diagnosis of a retroperitoneal benign cyst, likely a cystic lymphangioma, was established. Due to the size of the mass, a 5-mm port was added, and the cystic volume was reduced using a fine needle. Approximately 500 ml of clear yellow cystic fluid was aspirated. To prevent spillage of the cyst content during the procedure, the puncture hole was sealed with electrocoagulation. Notably, significant adhesions were observed between the lesion in the left anterior pararenal space (Figures 2B,C) and the aorta ventralis (Figure 2E), inferior vena cava (Figure 2E), and left renal pedicle (Figure 2D). This scenario led to the consideration of a macrocystic cystic lymphangioma. Consequently, complete resection with cystectomy was performed (Figure 2F). The operation lasted 235 min, with about 20 ml of intraoperative bleeding. Postoperatively, there were no incidents or complications, and the patient fully recovered within 10 days and was discharged.

**Figure 3:**
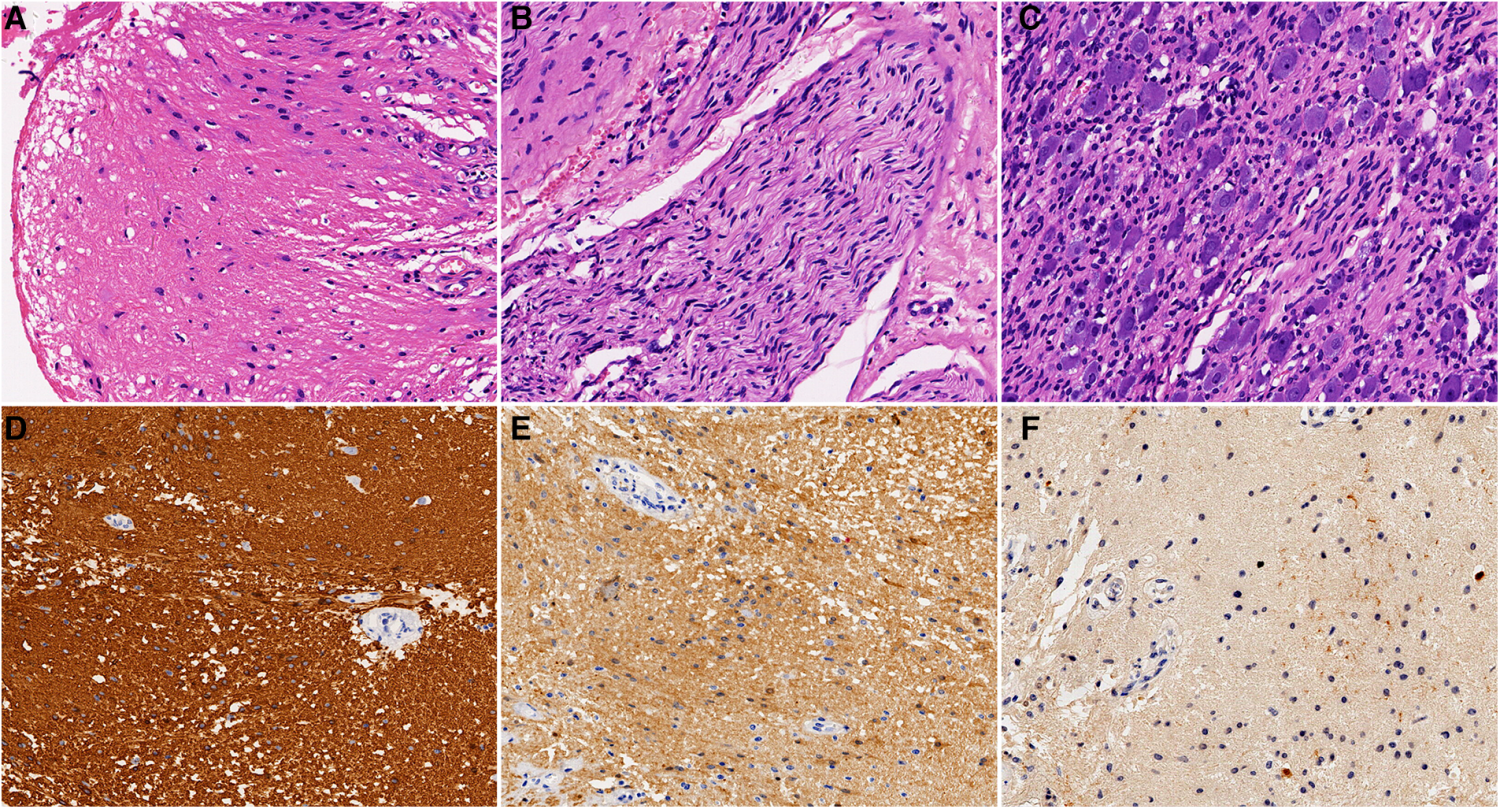
Postoperative pathology: (**A**) glial tissue (H&E, ×200). (**B**) Nerve bundle tissue (H&E, ×200). (**C**) Ganglia tissue (H&E, ×200). (**D**) Glial tissue was strongly positive for GFAP (IHC, ×200). (**E**) Glial tissue and nerve bundles were positive for S100 (IHC, ×200). (**F**) Glial tissue was weakly positive for NSE (IHC, ×200).

The resected thick-walled retroperitoneal cyst measured 120 mm × 98 mm. Histologically, the cyst wall consisted of fibrous connective tissue, mature central neuroepithelium (Figure 3A), nerve bundles (Figure 3B), and ganglia (Figure 3C). The neuroepithelium predominantly consisted of astrocytes with round or ovoid nuclei. The cytoplasm contained glial filaments, and there was no evidence of nuclear anisotropy or nuclear schizophrenia. Immunohistochemistry (IHC) confirmed strong positive expression of glial tissue using glial fibrillary acidic protein (GFAP) (Figure 3D). S100 also exhibited positivity in both the glial tissue and nerve bundles (Figure 3E). Weak positive expression of NSE was observed in the glial tissue, while positive expression was seen in the nerve bundles and ganglia (Figure 3F). No other embryonic derivatives or indications of malignancy were detected. These findings strongly support a diagnosis of cystic neuroglial heterotopia.

A 2-year follow-up ultrasound evaluation showed no signs of relapse. Further imaging modalities such as CT and MRI were not pursued due to the absence of positive findings on ultrasonography and limited financial resources. The parents expressed satisfaction with the outcomes of the laparoscopic treatment, citing reduced postoperative pain and minimal scarring as key benefits.

## 3 Discussion

Neuroglial heterotopia is a rare congenital developmental malformation first reported in the nasal cavity by Reid in 1852 (2). Over time, various terms have been introduced to describe this condition, including glial heterotopia, glial choristoma, heterotopic glial tissue, heterotopic neural tissue (HNT), ectopic brain, and ectopic neural tissue. Currently, “neuroglial heterotopia” stands as the widely accepted term among experts and scholars.

On the basis of anatomical site and pathological differentiation, neuroglial heterotopia is classified into paraneuraxial and extraneuraxial groups, with the latter being more commonly encountered (10). Paraneuraxial neuroglial heterotopia, characterized by a well-formed layer of brain tissue histologically, can occur in the paracranial region (occipital bone, deep cervical part) or paravertebral spaces (retroperitoneum) (8–10, 13). In contrast, the extraneuraxial group primarily affects the superficial layer of the head and neck, including the cavum nasi, and is characterized by the disorganization of neuroglial and mesenchymal tissues (2). The etiopathogenesis of retroperitoneal neuroglial heterotopia remains unclear. Several theories have been proposed, including (1) encephalocele and myelomeningocele occurring through a primary osseous defect followed by partial or complete secondary closure, resulting in sequestration of the herniated lesion (8–10); (2) retention of vestigial neural crest (3); (3) aberrant migration and differentiation of neuroectodermal remnants (14); and (4) hamartoma (6). However, none of these theories alone provide a satisfactory explanation for the pathogenesis.

The data pertaining to published cases of retroperitoneal neuroglial heterotopia are presented in Table 1. Among the five cases, a connection between the retroperitoneal lesion and the spine via a fibrous stalk was observed in an aborted fetus and a 3-year-old boy (9, 10), supporting the “herniation and sequestration” theory. However, this specific anatomical distribution was not identified in other cases. Fetal cases were associated with congenital anomalies such as facial dysmorphism, abdominal wall defects, and Mayer–Rokitansky–Küster–Hauser (MRKH) syndrome (10, 11). Typically, unilateral involvement of the retroperitoneum is observed, with a unifocal solid, soft, or cystic mass that compresses contiguous structures. With the exception of one case presenting urinary tract symptoms, most children may remain asymptomatic.

**Table 1:** Reported cases of retroperitoneal neuroglial heterotopia.

Year	Age/sex	Associated malformation	Affected side	Relationship with spine	Symptom	Type of lesion	Surgery/outcome
1992 (8)	6 years, female	No	Left retroperitoneum	Unconnected	Urinary tract symptoms	Cysts	Laparotomy, alive, no recurrence
1997 (9)	3 years, male	No	Right retroperitoneum	Cordlike stalk	Asymptomatic	Solid-cystic	Laparotomy, alive, no recurrence
1998 (10)	Abortion at 15 weeks of gestation, male	Facial dysmorphism, abdominal wall defect, small intestine, and colon eventration	Not described	Elongated string	—	Solid nodule	Death
2013 (11)	Induced labor, 14 weeks of gestation, female	MRKH syndrome	Right retroperitoneum, retropharyngeal space	Unconnected	—	Liquefied mass	Death
2017 (12)	3 months, male	No	Left retroperitoneum	Unconnected	Asymptomatic	Solid-cystic	Diagnosed by FNA, laparotomy, alive, no recurrence
Present	3 years, female	No	Left retroperitoneum	Unconnected	Asymptomatic	Cysts	Laparoscopy, alive, no recurrence

Urinary tract symptoms included urinary frequency, urgency, urge incontinence, and nocturnal enuresis.

While the preoperative serum NSE level was elevated in our case, this phenomenon was not observed in other cases (1–15). We explored the potential correlation between this elevation in NSE and the size of the mass, considering our case is the largest documented to date. Moreover, serum NSE testing was omitted during the follow-up period. Immunohistochemically, NSE exhibited positive expression, aligning with findings in similar cases (5, 7, 11, 15). Histologically, neuroglial heterotopia is characterized by mature astrocytes and glial fibers within a fibrovascular stroma, along with rare components like neurons, choroid plexus, and oligodendrocytes. Immunohistochemical staining for GFAP, S100, and NSE corroborated the presence of glial tissue in the current case. Additional markers such as synaptophysin (2, 5, 6, 10, 12, 14), NeuN (7), vimentin (5, 9, 14), chromogranin A (CGA) (2), CD56 (11), and neurofilament (NF) (5–7) contribute to confirming the diagnosis. It is imperative to further validate serum NSE or alternative biomarkers as diagnostic and prognostic tools for neuroglial heterotopia, necessitating a larger sample size to bolster their significance.

The CT imaging revealed a large, well-circumscribed, low-density solid-cystic or cystic mass. Furthermore, CT scans can provide information about the lesion's position, its relationship with surrounding normal structures, and any bony defects in the spine, which could have been further elucidated by MRI, although MRI was not conducted in our case. In light of the elevated NSE in our case, it is critical to distinguish cystic neuroblastoma. Ultrasonographically, cystic neuroblastoma typically presents as a hypoechoic mass that is either purely cystic or cystic-solid. It often exhibits prominent blood flow signals within the mass and may occasionally display small calcifications (16). CT imaging reveals an irregular cystic mass with thick walls, uneven wall thickness, or central enhancement. Cystic neuroblastoma is associated with an aggressive profile, commonly causing displacement or encasement of retroperitoneal vessels without invasive behavior, infiltrating paraspinal muscles and neural foramina, and leading to regional lymph node and liver metastases (16). Furthermore, cystic neuroblastoma typically manifests in infants younger than 1 year of age, most frequently affecting the adrenal glands. However, in our case, there were no corresponding imaging features indicative of cystic neuroblastoma. If imaging findings are inconclusive, further investigations such as a bone marrow smear or biopsy, a 24-h urine vanillylmandelic acid test, or tissue biopsy should be considered for additional evaluation. However, only one case underwent a diagnostic fine-needle aspiration (FNA) (12). Previous studies suggest that biopsies are not recommended for congenital midline lesions with potential intracranial connections due to the risk of cerebrospinal fluid leakage (2). Nevertheless, if an intracranial connection is excluded through MRI, a biopsy may be feasible, especially for ruling out malignancy. This approach, along with MRI, can be performed simultaneously in pediatric patients under anesthesia. Cytology analysis demonstrates masses of the fibrillary matrix corresponding to white matter, glial GFAP-positive cells, and cells with neuronal morphology. Intraoperative cytological examination of the cyst can assist in rapidly determining its nature. However, in the present case, neither intraoperative nor postoperative cytology was conducted. Despite various imaging examinations, the actual origin and precise preoperative diagnosis could not be determined. As a result, the final diagnosis primarily relies on histopathological examination.

The primary and most effective treatment for retroperitoneal neuroglial heterotopia is surgical intervention, which includes laparotomy and laparoscopy. Traditional open abdominal surgery offers adequate exposure and a clear view of the adjacent anatomical structures and spine displacement (8, 9, 12). This report describes the initial case of transperitoneal laparoscopic resection of a retroperitoneal mass. Several key points merit attention. First, employing suction decompression of cystic lesions can enhance the operative field for better visualization of neighboring organs. However, the conventional surgical principle of avoiding intraoperative rupture of any tumor is indisputable. Therefore, the decision to puncture should be made carefully, with a potential transition to laparotomy if necessary. Subsequently, the puncture site must be meticulously closed using electrocoagulation, suturing, and hemoclips to prevent any spillage. In addition, consideration should still be given to converting to laparotomy in cases where there are doubts about the nature of the mass, the presence of malignant cells in rapid cystic cytology, or difficulty in separating the cysts from adjacent structures. Second, precise delineation between retroperitoneal vessels and the lesion is crucial for successful radical resection. Without compromising the integrity of the cyst walls, the vascular skeletal anatomy enables the complete removal of the lesion while preventing critical vascular injury (17). Third, the preoperative placement of a ureteric double J stent can be particularly beneficial in cases complicated by hydronephrosis as it aids in identifying the ureter during laparoscopic procedures.

## 4 Conclusion

Retroperitoneal cystic masses with slow growth are uncommon in children and typically benign. The differential diagnosis encompasses various conditions, including adrenal cysts, cystic teratomas, bronchogenic cysts, Müllerian duct cysts, tailgut cysts, and lymphangiomas, among others. Neuroglial heterotopia, a less common variant of a rare form, should be considered in cases of cystic retroperitoneal masses. This presented case may enhance understanding of the pathogenesis, clinical characteristics, and treatment of such conditions. Laparoscopic excision emerges as a safe and viable therapeutic approach for retroperitoneal neuroglial heterotopia.

## Data Availability

The original contributions presented in the study are included in the article/Supplementary Material; further inquiries can be directed to the corresponding author.

## References

[1] Ali MJ Kamal S Vemuganti GK Naik MN. Glial heterotopia or ectopic brain masquerading as a dacyrocystocele. Ophthalmic Plastic Reconstr Surg. (2015) 31(2):e26–8. 10.1097/iop.0000000000000050 24492734

[2] Gallego Compte M Menter T Guertler N Negoias S. Nasal glial heterotopia: a systematic review of the literature and case report. Acta Otorhinolaryngol Ital. (2022) 42(4):317–24. 10.14639/0392-100x-n1977 36254649 PMC9577684

[3] Shim HJ Kang YK An Y-H Hong YO. Neuroglial choristoma of the middle ear with massive tympanosclerosis: a case report and literature review. J Audiol Otol. (2016) 20(3):179–82. 10.7874/jao.2016.20.3.179 27942605 PMC5144819

[4] Alonso L Sevilla J Gonzalez-Vicent M Abad L Gonzalez-Mediero I Diaz MA. Pulmonary glial heterotopia in a child diagnosed with Fanconi anemia and epilepsy. J Pediatr Hematol Oncol. (2011) 33(6):462–4. 10.1097/MPH.0b013e318215cef0 21792042

[5] Buccoliero AM Caldarella A Noccioli B Fiorini P Taddei A Taddei GL. Brain heterotopia in pharyngeal region. A morphological and immunohistochemical study. Pathol Res Pract. (2002) 198(1):59–63. 10.1078/0344-0338-00186 11866213

[6] Price KM Cummings TJ Etter JR Woodward JA. Ectopic brain in the orbit presenting as disc edema in an adult. Orbit. (2009) 28(1):74–7. 10.1080/01676830802599150 19229751

[7] Glavis-Bloom J Nahl D Rubin EM Nael A Dao T. Congenital neuroglial choristoma of the foot. Radiol Case Rep. (2019) 14(6):718–22. 10.1016/j.radcr.2019.03.025 30988863 PMC6447744

[8] Wan J Ritchey ML Muraszko K Bloom DA. Retroperitoneal neurogenous choristoma. J Urol. (1992) 148(6):1867–8. 10.1016/s0022-5347(17)37052-0 1433623

[9] Hori A Brandis A Walter GF Petersen C Massmann J. Retroperitoneal ectopic neural mass: “abdominal brain”—presentation of two cases and proposal of classification of paraneuraxial neural ectopia. Acta Neuropathol. (1998) 96(3):301–6. 10.1007/s004010050898 9754964

[10] Hahlbohm AM Hori A Hoyer PF Petersen C. Ectopic neural tissue as an unusual cause of a retroperitoneal tumor. Pediatr Surg Int. (2013) 12(1):66–8. 10.1007/bf01194808 9035216

[11] Deng LH Lee CH. Multicentric paraspinal neuroglial heterotopia with Müllerian and renal agenesis: a variant of Mayer-Rokitansky-Küster-Hauser syndrome? Diagn Pathol. (2013) 8(1):141. 10.1186/1746-1596-8-141 23968558 PMC3849052

[12] Díaz del Arco C Ortega Medina L Subhi-Issa Ahmad I. Retroperitoneal ectopic brain: case report and literature review. Diagn Cytopathol. (2017) 46(6):528–31. 10.1002/dc.23882 29280334

[13] Aboud MJ. Respiratory difficulty caused by an ectopic brain tissue mass in the neck of a two-month-old baby: a case report. J Med Case Rep. (2011) 5(1):220. 10.1186/1752-1947-5-220 21649939 PMC3118211

[14] Arndt S Wiech T Mader I Aschendorff A Maier W. Rare extracranial localization of primary intracranial neoplasm. Diagn Pathol. (2008) 3:14. 10.1186/1746-1596-3-14 18416840 PMC2330022

[15] Arredondo Montero J Anaut MB Pascual CB. Unilateral anophthalmia and congenital frontal cranioschisis associated with extradural neuroglial heterotopia: new insights into a possible new malformative spectrum. Fetal Pediatr Pathol. (2023) 42(2):275–80. 10.1080/15513815.2022.2086330 35670570

[16] Eo H Kim JH Jang KM Yoo SY Lim GY Kim MJ Comparison of clinico-radiological features between congenital cystic neuroblastoma and neonatal adrenal hemorrhagic pseudocyst. Korean J Radiol. (2011) 12(1):52–8. 10.3348/kjr.2011.12.1.52 21228940 PMC3017884

[17] Liu Z Xiao Y Chen D Wang Z. Vascular skeletalization: a new concept to improve the resection rate in childhood neuroblastoma. J Neurosurg Sci. (2014) 58(2):113–6. 24819488

